# Preclinical Intracranial Aneurysm Models: A Systematic Review

**DOI:** 10.3390/brainsci10030134

**Published:** 2020-02-27

**Authors:** Fabio Strange, Basil E Grüter, Javier Fandino, Serge Marbacher

**Affiliations:** 1 Department of Neurosurgery, 5001 Kantonsspital Aarau, Switzerland; basil.grueter@ksa.ch (B.E.G.); javier.fandino@ksa.ch (J.F.); serge.marbacher@ksa.ch (S.M.); 2 Cerebrovascular Research Group, Department for BioMedical Research, University of Bern, 3008 Bern, Switzerland

**Keywords:** animal model, aneurysm, cerebral aneurysm, intracranial aneurysm

## Abstract

Intracranial aneurysms (IA) are characterized by weakened cerebral vessel walls that may lead to rupture and subarachnoid hemorrhage. The mechanisms behind their formation and progression are yet unclear and warrant preclinical studies. This systematic review aims to provide a comprehensive, systematic overview of available animal models for the study of IA pathobiology. We conducted a systematic literature search using the PubMed database to identify preclinical studies employing IA animal models. Suitable articles were selected based on predefined eligibility criteria following the Preferred Reporting Items for Systematic Reviews and Meta-Analyses (PRISMA) guidelines. Included studies were reviewed and categorized according to the experimental animal and aneurysm model. Of 4266 returned results, 3930 articles were excluded based on the title and/or abstract and further articles after screening the full text, leaving 123 studies for detailed analysis. A total of 20 different models were found in rats (nine), mice (five), rabbits (four), and dogs (two). Rat models constituted the most frequently employed intracranial experimental aneurysm model (79 studies), followed by mice (31 studies), rabbits (12 studies), and two studies in dogs. The most common techniques to induce cerebral aneurysms were surgical ligation of the common carotid artery with subsequent induction of hypertension by ligation of the renal arteries, followed by elastase-induced creation of IAs in combination with corticosterone- or angiotensin-induced hypertension. This review provides a comprehensive summary of the multitude of available IA models to study various aspects of aneurysm formation, growth, and rupture. It will serve as a useful reference for researchers by facilitating the selection of the most appropriate model and technique to answer their scientific question.

## 1. Introduction

Intracranial aneurysm (IA) refers to an outward bulging of the arterial wall and is a serious cerebrovascular disease with a high morbidity and mortality [1]. It is characterized by a chronic inflammation and weakening of the arterial walls [2]. The prognosis of IA is poor, due to a rupture of the lesions and the ensuing subarachnoid hemorrhage that is responsible for the high number of IA-induced fatalities. Even though the prevalence of IA is high (2–8% [3]), there is currently no proven therapy that achieves stabilization and prevention of rupture. Most IA patients are treated conservatively, and only those with a presumably high risk of IA rupture (depending on the IA size, smoking status and location [4]) undergo occlusion [5]. The successful development and implementation of therapeutic strategies to avoid IA formation, and particularly subarachnoid hemorrhage, is hence of clinical importance. A prerequisite for any effective therapy is a better understanding of IA pathobiology. Moreover, both the efficacy and potential side effects of a novel drug need to be carefully assessed before it may be administered to IA patients, requiring a thorough preclinical investigation that precedes the translation in the clinical practice. Since the natural formation of IA is rare in animals, techniques to artificially induce IA in experimental animals have been developed. Researchers interested in the study of IA pathobiology are now facing a broad variety of animal models to choose from [6]. These involve models in different species and numerous variations of the originally developed methods, which differ in their comparability to human IAs, the complexity of the methodology, and the questions that can be answered. Furthermore, the technique to induce hypertension constitutes a common variation of the initial, well-established models. The large volume of available models may complicate the selection of the appropriate model for the respective research question. We therefore set out to compile systematic literature review on available IA animal models as a comprehensive reference for researchers planning to employ such a model in their investigations. We discuss advantages and disadvantages of each model and address considerations regarding the species and method of choice.

## 2. Materials and Methods

A systematic literature search in the Medline/PubMed database was conducted to identify preclinical studies using IA animal models. The search was performed on November 31, 2017 with the keywords “mice”, “rat”, “rabbit”, “dog”, and “swine” in combination with “aneurysm” using the Boolean operator [AND]. Studies on primates were excluded due to their limited ethical justifiability. The search was restricted to “animals”. Two investigators (SM and FS) independently screened titles and abstracts and selected suitable studies based on predefined eligibility criteria following the Preferred Reporting Items for Systematic Reviews and Meta-Analyses (PRISMA) [7]. The final articles to be included were selected based on the full text of eligible studies. Discrepancies in the study selection were discussed with all authors and a consensus was reached. Included studies were reviewed and categorized according to the experimental animal used and the aneurysm model employed.

The eligibility criteria were as follows: (1) in vivo IA model in the experimental species rat, mouse, rabbit, dog, and swine; (2) English language; (3) original research article (reviews, letters, and editorials were excluded).

The following data were extracted from eligible full text articles: (1) authors and year of publication; (2) study question and main conclusion; (3) animal species; (4) method to create IA; (5) IA location; and (6) time until sacrifice/study duration. 

The same method to generate IA in different species was considered a separate model. Modifications of an existing model such as extension of the technique itself or of significant accessory techniques (e.g., induction of hypertension by renal artery (RA) ligation) were also categorized as individual models. 

## 3. Results

**Figure 1:**
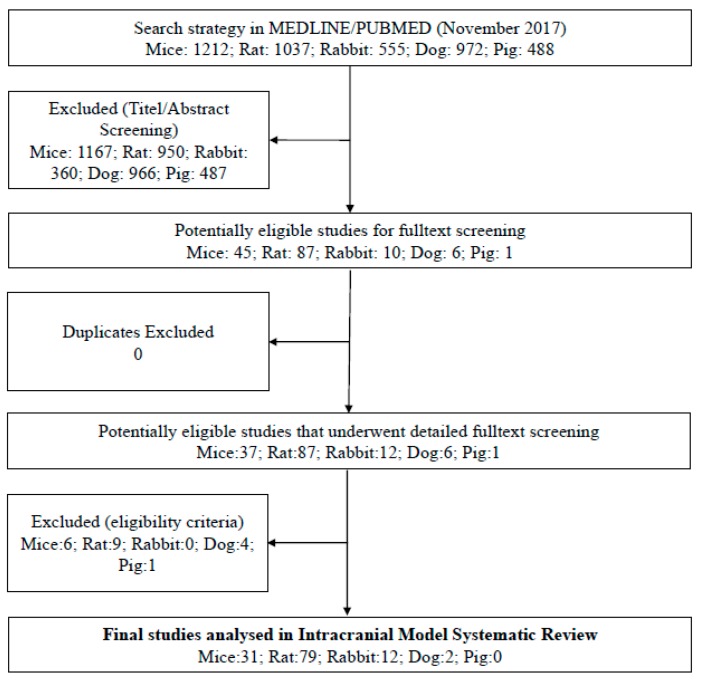
Preferred Reporting Items for Systematic Reviews and Meta-Analyses (PRISMA) flow chart for study selection.

The literature searches initially returned 4264 articles, of which 3930 were excluded after title and/or abstract screening because they did not meet the eligibility criteria. A further 211 articles were excluded after screening the full text due to one or more of the following reasons: duplicate article, article was withdrawn, the type of article differed from an original research study such as a review, letter or comment, or the article was written in a language other than English. This strategy left 123 studies for a detailed analysis (Figure 1).

A total of 20 different models were identified based on the technique to create IA (common carotid artery (CCA) ligation (unilateral or bilateral), (renal artery [RA] ligation [unilateral or bilateral], elastase injection [with or without CCA ligation] and experimental species [rat, mouse, rabbit, dog])) (Table 1). Only those modifications that affected the ligation surgery (i.e., RA ligation or not) were considered as separate models. Smaller variations to the induction of hypertension (i.e., different NaCl concentrations, β-aminopropionitrile monofumarate (BAPN) or estrogen depletion) were not considered as novel models. Of the identified distinct models, 9 different models were used in rats, 5 in mice, 4 in rabbits, and 2 in dogs (see Table 1 for detailed listing of studies according to the IA model). The identified IA animal models were assigned to one of the following main categories: (1) CCA ligation with concomitant RA ligation, (2) CCA ligation only, (3) elastase injection, (4) elastase injection and CCA ligation, or (5) another model.

The most common technique to induce cerebral aneurysms was surgical ligation of the CCA and/or the RA with concomitant induction of hypertension (64 studies), followed by CCA ligation without RA manipulation (24 studies) and elastase-induced creation of IAs in combination with corticosterone- or angiotensin II-induced hypertension (24 studies, see Table 2 for an overview of IA model by experimental animal). Thirteen studies employed alternative methods to create IA. Of the models using CCA ligation, unilateral ligation of the left CCA was performed in most models, but ligation of the right CCA or bilateral ligation was also common. CCA ligation was most common in rats and rabbits, whereas the elastase method was used in most mouse models. The category “other models” included IA creation by deoxycorticosterone administration [121], eplerenone administration [122], induction of copper deficiency [123], CaCl_2_ treatment [124], coating of the internal carotid artery [125], with one study for each of these models.

Most of the assessed studies used rats for their animal model (79 studies), followed by mice (31 studies), and rabbits (12 studies). Only two studies were performed in dogs. Almost half of the mouse models were employed in transgenic animals [22,41,43,53,92,95,96,99,102,105,106,109,112,114,127].

The most frequent variation of the original models was modification of the technique to induce hypertension rather than of the IA creation technique itself. Hypertension was typically achieved by using one or a combination of RA ligation, high salt diet, or deoxycorticosterone administration (Table 3). A further variation of the original CCA model was omission of BAPN administration to inhibit cross-linking of collagen and elastin, which was contraindicated depending on certain research questions [56].

The study duration before sacrifice and assessment of the animal varied between a few weeks [38,105] and a year [16,71], but typically lasted 1 to 3 months.

## 4. Discussion

By conducting a comprehensive systematic review of the literature, we achieved categorization of the IA animal models available to date and developed an overview that should facilitate the choice of the experimental animal and most appropriate technique for researchers interested in IA pathobiology. Surgical ligation of the CCA and elastase-induced weakening of the arterial vessel wall was identified as the predominant techniques to create IA in experimental animals. A brief discussion of the advantages and disadvantages of each of these categories follows.

### General Considerations

Ethics: Animal ethics regulations have become increasingly strict in recent years in terms of the species of animal and required numbers to be used in experimental studies. Studies on animals that raise particular ethical concerns, such as those conducted in dogs, primates and swine, are likely to face more obstacles throughout the animal ethics approval process, which may delay the conduction of the study or entirely prevent its completion. Small animals that are more commonly used in research such as rats and mice appear often more feasible than those in large animals, yet the study needs to be carefully designed to ensure statistically feasible justification of the required animal numbers, particularly in cases where it is unknown if and for how long the animals will survive the experiment, as is the case for all intracranial animal models.

Costs: Similar to the ethical concerns, the maintenance and housing of large animals can infer tremendous costs on the researcher. In addition, the potential costs need to consider the duration of the experiments, as some of the aneurysm models last for more than three months, during which time the animals need to be housed and likely monitored daily, which results in additional costs for animal care personnel and veterinarians.

Reliability: Most intracranial aneurysm models are conducted in rats by unilateral CCA ligation and concomitant RA ligation (Table 2). This can be attributed to the reliability of such a model in producing aneurysms in the majority of experimental animals in a comparatively very short period of time of less than three months (Table 3). These models are so reliable because they are based on a long history of refinement and modifications, Table S1 tweaking the technique to a point where aneurysm development is guaranteed. An even faster generation of aneurysms is observed in the elastase model in mice, yet it appears that this model does not work well in rats, therefore one needs to balance the time of formation with the most suitable species for the experiment. The best model clearly depends on the research question to be answered. If aneurysm growth is the main factor to be investigated, surgical ligation models are to be favored over others, as the procedure allows for variation in the size of the aneurysm and may generate very large aneurysms in the animals. In turn, if aneurysm rupture is the center of the investigation, the surgical model needs to be combined with a treatment to induce hypertension such as administering NaCl in the drinking water or implanting a salt pellet with dosed release. Alternatively, the elastase model is frequently used in mechanistic and pharmacological aneurysm rupture studies as it generates ruptures through subarachnoid hemorrhages within a relatively short period of time (one month).

Ligation of the CCA, with or without concomitant RA ligation: This original model first described in the 1970s by Hashimoto et al. [11] and Nagata et al. [78] involves unilateral ligation of the CCA, for which most frequently the left CCA is manipulated. The ligation is accompanied by additional induction of hypertension using either ligation of one or both RAs, feeding of a high salt diet, administration of deoxycorticosterone, or a combination of these parameters (Table 4). In order to increase the animal’s susceptibility to IA formation and shortening of the IA induction time, CCA ligation may be combined with administration of β-aminopropionitrile, a lathyrogen that inhibits the cross-linking of collagen and elastin, or with estrogen depletion achieved by oophorectomy to compromise endothelial cell function and NO (nitrogen oxide) release. Most rat models employ unilateral CCA ligation combined with bilateral RA ligation and a high salt diet, while in rabbits, both CCAs are often ligated without any further means of inducing hypertension. In mice, unilateral CCA ligation was complemented by contralateral ligation of one RA.

The major advantage of this model is that is has been well established and refined for decades and has been shown to reliably induce IA in rats, mice and rabbits. It has been employed to answer a broad variety of research questions, ranging from the contribution of isolated factors to aneurysm formation to the success of therapeutic agents in the prevention and treatment of IAs.

Elastase treatment: As an alternative to the classical vessel ligation model, several more recent studies employ the injection of elastase into the cerebrospinal fluid or a common artery to disrupt the elastic lamina and thereby weaken the vessel walls. Concomitant induction of hypertension is achieved by continuous administration of angiotensin II with the aid of an implanted osmotic mini-pump, or by a combined administration of a high salt diet and deoxycorticosterone. This model is employed in mice and rabbits but rarely in rats. It accomplishes creation of large aneurysms within a relatively short study period. In a few models, both the injection of elastase and artery ligation were performed. 

**Figure 2:**
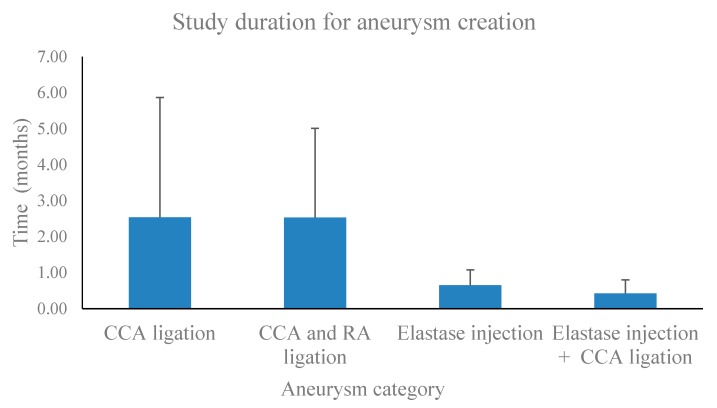
Meta-analysis of all intracranial aneurysm models to determine the shortest required study period for intracranial aneurysm (IA) generation. Common carotid artery (CA) ligation alone or in combination with renal artery (RA) ligation typically involved a period of several months, while intracranial aneurysms could be created by elastase injection with or without common carotid artery ligation in one month or less. Bars indicate mean + SD.

Elastase treatment and ligation of the CCA: IA generation by elastase injection may be supported by concomitant ligation of the CCA. We identified only four studies that employed this model, and mice were the only experimental animal used. Hence, this model has apparently not been tested in other experimental animals. Of note, this model involved the shortest study period, with IA creation achieved several days following the induction and a mean generation time of less than half a month (Figure 2, Table 3). 

Once the type of IA model has been identified, the choice of experimental animal species must be considered, considering that the situation in the animal should be comparable to human IA pathophysiology. Most IA models are performed in small animals such as rats and mice. A limitation of all models is that IA do not easily develop spontaneously in animals and hence always constitute an artificially induced entity.

Certain models apparently work better in one species than the other [25]. The total study duration needs to be estimated; e.g., how long does it take to induce an aneurysm in an animal, and is this duration feasible for the study setting? We performed a meta-analysis of all selected studies with regards to the time it took to develop IAs following the induction (Figure 2, Table 3). Elastase injection alone (0.65 ± 0.43 months), or in combination with CCA ligation (0.43 ± 0.38 months), required a shorter time for IA generation than CCA alone (2.53 ± 3.33 months) or in combination with RA ligation (2.38 ± 2.48 months; Figure 2). The shortest study period was observed in a mouse model with elastase injection (0.1 month), while a model with CCA ligation in mice (13 months) took the longest (Table 3).

In addition, the number of animals that can be used is determined by the breeding time, housing space and animal care cost, and is to be carefully considered to obtain an animal number that can reach statistical significance (Collaborative Approach to Meta-Analysis and Review of Animal Data from Experimental Studies/CAMARADES [128]). Furthermore, different strains of the same species may differ in their susceptibility to IA formation [36]. Practical aspects also play a role in the species choice, such as the space that is required for the surgery (e.g., can it be done in a designated laboratory space or is an operating theater required) and the level of difficulty of the surgery itself (e.g., is an anesthesiologist required, how technically challenging is the surgery). The estimation of whether the respective research question can be appropriately answered with the experimental animal species is of utmost importance. For example, if the contribution of an isolated factor is to be investigated, a strain with a genetic knockdown of the gene of choice may be useful, which then renders a genetic mouse model the most suitable choice. Animal ethics need to be considered as well, as canine studies are not only expensive but also ethically challenging. Concerns over the ethical treatment of animals led to the development of the “3R principle” that aims to replace, reduce, or refine experiments using experimental animals; owing to this principle, primates are rarely used these days as research models. For this reason, we did not extend the scope of the present review to this species as they are only employed in exceptional cases. Advantages and disadvantages of each model are discussed as follows.

Rats: The major advantage of using rats as an experimental model is the availability of a well-established model that works well and has been described in detail with slight variations for decades. CCA ligation in combination with RA ligation appears to be the gold standard for IA creation, and the investigator can make a well informed choice of the experimental specifications due to the plethora of available literature. Furthermore, rats are commonly used experimental animals, are easily available, instill only moderate costs for housing and feed, and breed fast, making it possible to conduct studies with many animals to reach statistical significance. Moreover, anesthetic techniques are well established in this species and can be easily maintained in a typical research laboratory without the need for a designated operating theater or a veterinary anesthesiologist. The surgery is manageable (not as small as in the mouse but still comparable to humans) and may be relatively easily learned by a new scientist entering the field. The disadvantage of using rats is that the elastase model apparently does not work as well in rats as in mice, and neither does bilateral CCA ligation should it be required. In addition, although genetic knockout models in rats are available, they generate considerably higher costs compared to such models in mice.

Mice: Mice share the same practical advantages as rats, in that their housing and feed is comparatively cheap, they breed fast, and are well maintained during surgery on basic anesthetic techniques. The major advantage of using mice as an experimental model is the possibility to investigate an isolated factor that may contribute to IA formation or protect from it in transgenic animals, be it knockout mouse models or animals overexpressing certain genes. Particularly, the elastase model is well established in mice, and the CCA ligation model has also been successfully employed in this species. A disadvantage of using mice may be the somewhat delicate surgery on very small vessels, which could require some practice to achieve an optimum outcome. 

Rabbits: Bilateral CCA ligation is particularly well established in rabbits and appears to work better in this species than in rats. None of the studies included in this review employed RA ligation in rabbits, and only one study described the elastase model. In terms of practicability, rabbits incur a somewhat larger cost for housing than rats and mice as they require more space. It may also not be possible to conduct surgery on these animals in any regular laboratory space, but rather an operating theater might be required. 

Dogs: We identified only two studies using distinct IA models in dogs as experimental animals, both of which were dated (1977 and 1984). It has to be noted that angiography is an investigative technique available for dogs that does not require sacrifice of the animal. Nevertheless, it appears that both the much more extensive cost in combination with ethical considerations does not render this species a model that can be routinely employed. The same holds true for swine and primates as experimental animals to create IAs. Few studies exist that use large species to produce IAs. The few published studies are considered historical series rather than models that are still in use today. Considering ethical concerns, it is unlikely that these species/models will gain importance in the future.

## 5. Conclusions

We provide a categorization of available IA animal models and thereby present a tool to guide researchers entering the field of aneurysm pathobiology. The best choice of a specific IA model strongly depends on the individual research question and numerous other factors such as the primary endpoint, available resources (e.g., expenses for animal housing and breeding, space for surgical procedures, need for veterinary anesthesiologist), and time frame for IA initiation, growth, and rupture (weeks to months). 

## Figures and Tables

**Table 1:** Overview of included studies. The most commonly used model in rats was common carotid artery (CCA) ligation and renal artery (RA) ligation, in mice elastase injection, and in rabbits CCA ligation.

Category	Subcategory		References
CCA ligation	Unilateral CCA ligation		Rat: Alvarez et al. 1986 [8], Cai et al. 2012 [9], Coutard et al. 2000 [10], Hahimoto et al. 1978 [11], 1979a [12], 1979b [13], 1980 [14], Ishibashi et al. 2012 [15], Kaufmann et al. 2006 [16], Matsushita et al. 2012 [17], Suzuki et al. 1980 [18], Roda et al. 1988 [19], Xu et al. 2011a [20], 2011b [21]
			Mouse: Abruzzo et al. 2007 [22]
			Rabbit: Dai et al. 2013 [23], Gao et al. 2008 [24]
	Bilateral CCA ligation		Rat: Tutino et al. 2016 [25]
			Rabbit: Dolan et al. 2013 [26], Kolega et al. 2011 [27], Li et al. 2014 [28], Liaw et al. 2014 [29], Mandelbaum et al. 2013 [30], Metaxa et al. 2010 [31], Tutino et al. 2014 [32], 2015 [33]
CCA ligation and RA ligation	Unilateral CCA ligation	Unilateral RA ligation	Rat: Aoki 2007 [34], 2014 [35], Coutard et al. 1997 [36], Fukuda et al. 2014 [37], Ikedo et al. 2017 [38], Miyata et al. 2017 [39], Yamamoto et al. 2017 [40]
			Mouse: Aoki et al. 2007 [34], 2017, Moriwaki et al. 2006 [41]
			Rabbit: Gao et al. 2008 [24]
		Bilateral RA ligation	Rat: Alvarez and Roda 1986 [8], Aoki et al. 2007a [42], 2007b [43], 2008a [44], 2008b [45], 2008c [46], 2008d [47], 2009 [48], 2010a [49], 2010b [50], 2011 [51], 2012 [52], 2017a [53], 2017b [54], Eldawoody et al. 2009 [55], Futami et al. 1995a [56], 1995b [57], 1998 [58], Guo et al. 2016 [59], Hazama et al. 1986 [60], Ishibashi et al. 2010 [61], Jamous et al. 2005a [62], 2005b [63], 2005c [64], 2007 [65], Kang et al. 1990 [66], Kim et al. 1988 [67], Kim et al. 1993 [68], Kimura et al. 2010 [69], Kojima et al. 1986 [70], Kondo et al. 1997 [71], 1998 [72], Korai et al. 2016 [73], Li et al. 2014 [74], 2015 [75], Maekawa et al. 2017 [76], Miyamoto et al. 2017 [77], Nagata et al. 1979 [78], 1980 [79], 1981 [80], Nakatani et al. 1993 [81], Sadamasa et al. 2007 [82], 2008 [83], Tada et al. 2010 [84], 2011 [85], Tamura et al. 2009 [86], Yagi et al. 2010 [87], Yamazoe et al. 1990 [88], Yokoi et al. 2014 [89], Wu et al. 2016 [90], Zhou et al. 1985 [91]
			Mouse: Sadamasa et al. 2003 [92]
Elastase injection			Rat: Zhao et al. 2015 [93]
			Mouse: Chalouhi et al. 2016 [94], Chu et al. 2015 [95], Hasan et al. 2015 [96], Kanematsu et al. 2011 [97], Kuwabara et al. 2017 [98], Labeyrie et al. 2017 [99], Lee et al. 2016 [100], Liu et al. 2016 [101], 2017 [102], Makino et al. 2012 [103], 2015 [104], Nuki et al. 2009 [105], Pena Silva et al. 2014 [106], 2015 [107], Shimada et al. 2015a [108], 2015b [109], Tada et al. 2014a [110], 2014b [111], 2014c [112], Wada et al. 2014 [113], Zhang et al. 2015 [114]
			Rabbit: Dai et al. 2010 [115], Yasuda et al. 2005 [116]
Elastase injection and CCA ligation			Mouse: Hoh et al. 2014 [117], Hosaka et al. 2014 [118], 2017 [119], Nowicki et al. 2017 [120]
Other	Deoxycorticosterone/hypertension		Lee et al. 1978 [121] (rat)
	Eplerenone		Tada et al. 2009 [122] (rat)
	Copper deficiency		Jung et al. 2016 [123] (rat)
	CaCl2		Bo et al. 2017 [124] (rat)
	Coating of internal carotid artery		Ebina et al. 1984 [125] (dog)
	Venous pouch or venous patch		Nishikawa et al. 1977 [126] (dog)

**Table 2:** Number of aneurysm type per species in the included studies. The vast majority (76%) of studies in rats used a combined CCA ligation and RA ligation model, whereas most mouse studies (68%) used an Elastase only model. CCA = common carotid artery; RA = renal artery.

Species	Total No. of Studies Analyzed	CCA Ligation and RA Ligation	CCA Ligation Only	Elastase Only	Elastase and CCA Ligation	Other
Mice	31	4	1	21	4	-
Rats	79	60	13	1	-	5
Rabbits	21	-	10	1	1	-
Dogs	2	-	-	-	-	2
Overall	133	64	24	22	5	6

**Table 3:** Mean time of aneurysm formation in months (range).

Species	CCA Ligation and RA Ligation	CCA Ligation Only	Elastase Only	Elastase and CCA Ligation	Other
Mice (31)	3,4 (0,5–5)	13 (0)	0,53 (0,17–1)	0,43 (0,1–0,75)	-
Rats (79)	2,28 (0,17–12)	2,49 (0,25–12)	1,25 (0)	-	1,83 (1–2,5)
Rabbits (12)	3,0	2,59 (0,17–6)	1,5 (1–2)	-	
Dogs (2)	-	-	-	-	0,68 (0,35–1)
Overall	2,38 (0,17–12)	2,53 (0,17–13)	0,65 (0,17–2)	0,43 (0,1–0,75)	1,37 (0,35–2,5)

**Table 4:** Variations to established IA models pertaining to the technique to induce hypertension and weaken vessel walls.

Technique	Purpose	Number of Studies	Number of Species
RA ligation, unilateral	Hypertension	12	3
RA ligation, bilateral	Hypertension	52	2
High NaCl diet (1% or 8%)	Hypertension	68	3
Deoxycorticosterone administration	Hypertension	15	3
Angiotensin II	Hypertension	13	1
β-aminopropionitrile administration (0.12%)	Weakening of vessel walls	36	2
Estrogen depletion/oophorectomy	Weakening of vessel walls	12	3

Note: several methods may have been used concomitantly in the same animal or study. High NaCl diet followed by bilateral RA ligation were the most commonly used techniques. RA = renal artery; NaCl = sodium chloride.

## Supplementary Materials

The following are available online at https://www.mdpi.com/2076-3425/10/3/134/s1, Table S1: Incidence of Aneurysm development.
