# Supplementary material for: Preclinical Intracranial Aneurysm Models: A Systematic Review
Source: Brain Sci. 2020 Feb 27;10(3):134. doi: 10.3390/brainsci10030134 (PMC7139747; doi:10.3390/brainsci10030134)
Supplement: Supplementary file 1 [file brainsci-10-00134-s001.pdf]

Supplementary Table 1 – Incidence of Aneurysm development

**CCA ligation only**	**Species**	**Author Year**	**Incidence**
Unilateral	Mouse	Abruzzo et al 2007	2/2 (100%)
	Rat	Alvarez 1986	2/10 (20%)
		Cai et al 2012	3/13 (23%)
		Coutard et al 2000	LE 24% /F2 42% /BC 50%
		Hahimoto et al. 1978	2/5(40%)
		Hashimoto 1979a	11/30 (37%)
		Hashimoto 1979b	10%
		Hashimoto 1980	21/67(31.34%)
		Ishibashi 2012	100%
		Kaufmann 2006	15/82 (18.3%)
		Matsushita 2012	6/20 (30%) / 16/20(80%)
		Suzuki 1980	4/19(21%), 3/15(20%), 2/9(22%)
		Roda 1988	3/8 (37.5)
		Xu 2011a<br>Xu 2011b	100%
	Rabbit	Dai et al 2013	0%
		Gao et al 2008	88.75%
Bilateral	Rat	Tutino 2016	na
	Rabbit	Dolan et al 2013	na
		Kolega et al 2011	100%
		Li 2014	100%
		Liaw 2014	100%
		Mandelbaum 2013	100%
		Metaxa 2010	100%
		Tutino 2014<br>Tutino 2015	17-78%
	Primate	Tenjin 1995	100%
**CCA and RA ligation**			

Unilateral	Mouse	Aoki 2007	9/16 (56%) / 9/10(90%)
		Aoki 2014	15/22 (68.18%)
		Morikawi 2006	6/11 (54.55%)
	Rat	Aoki 2007	19/21 (90%)
		Aoki 2014	100%
		Coutard 1997	LE, 56%; Wistar, 33%; BN, 17%; LOU, 11%. Carotid ligation only, LE (86%) BN (7%)
		Fukuda 2014	>80%
		Ikedo 2017	na
		Miyata 2017	na
		Yamamoto 2017	90/92 (97.8%)
	Primate	Yamamoto 2017	-
		Hashimoto 1987	-
	Rabbit	Gao 2008	na
Bilateral	Mouse	Sadamasa 2003	10/16(62.5%) / 6/8 (74%)
	Rat	Alvarez 1986	6/9 (66.67%)
		Aoki 2007a	10/19(53%) / 19/21(91%)
		Aoki 2007b	
		Aoki 2008a	
		Aoki 2008b	19/21 (90%)
		Aoki 2008c	
		Aoki 2008d	
		Aoki 2009	na
		Aoki 2010a	
		Aoki 2010b	na
		Aoki 2011	100%
		Aoki 2012	na
		Aoki 2017a	na
		Aoki 2017b	
		Eldawoody 2009	100%

Futami 1995a	23/31(74.2%) / 10/16 (62.5%) / 37.5%
Futami 1995b	
Futami 1998	14/29 (48.28%)
Guo 2016	30/71 (42.25%)
Hazama 1986	8/22 (36.36%)
Ishibashi 2010	NA
Jamous 2005a	60-100%
Jamous 2005b	
Jamous 2005c	
Jamous 2007	
Kang 1990	8/11 (72.75%)
Kim 1988	10/12 (83.3%)
Kim 1993	na
Kimura 2010	50%
Kojima 1986	100%
Kondo 1997	
Kondo 1998	
Korai 2016	
Li 2014	
Li 2015	
Maekawa 2017	
Miyamoto 2017	6/27(22%) / 15/26 (58%)
Nagata 1979	29-100%
Nagata 1980	
Nagata 1981	
Nakatani 1993	15/20 (75%)
Sadamasa 2007	28/28(100%)
Sadamasa 2008	100%
Tada 2010	na
Tamura 2009	50%

		Yagi 2010	30%
		Yamazoe 1990	11/19 (57.89%)
		Yokoi 2014	na
		Wu 2016	27/30 (90%)
		Zhou 1985	na
**Elastase injection**			
	Mouse	Cgalouhi 2016	100%
		Chu 2015	90%
		Hasan 2015	>80%
		Kanematsu 2011	6/10 60%) / 14/20 (70%)
		Kuwabara 2017	82%
		Labeyrie 2017	na
		Lee 2016	8/11 (72.75%)
		Liu 2016	>90%
		Makino 2012	7/10 (70%)
		Makino 2015	6/10 (60%)
		Nuki 2009	77%
		Pena Silva 2014	89%
		Pena Silva 2015	15/16 (94%) / 12/14 (86%)
		Shimada 2015a	
		Shimada 2015b	88% / 19/23(82.3%)
		Tada 2011	
		Tada 2014a	60-80%
		Tada 2014b	
		Tada 2014c	
		Wada 2014	70–80%
		Zhang 2015	na
	Rat	Zhao 2015	41.70%
	Rabbit	Dai 2010	0%
		Yasuda 2005	100%

**Elastase and CCA**			
	Mouse	Hoh 2014	89%
		Hoasaka 2014	100%
		Hoasaka 2017	na
		Nowicki 2017	75%
**Others**			
Deoxycorticosterone/hypertension	Rat	Lee 1978	na
Eplerenone	Rat	Tada 2009	59%
Copper deficiency	Rat	Jung 2016	46.60%
CaCl2	Rat	Bo 2017	na
Coating of internal carotid artery	Dog	Ebina 1984	na
Venous pouch or venous patch	Dog	Nishikawa 1977	55%
